# Barriers to satisfactory primary care contacts for Swedish cancer survivors: a cross-sectional survey

**DOI:** 10.1080/02813432.2026.2701788

**Published:** 2026-07-11

**Authors:** Hanna Glock, Anna-Maria Larsson, Susanna Calling, Lars Garpenhag

**Affiliations:** a Center for Primary Health Care Research, Department of Clinical Sciences Malmö, Lund University, Malmö, Sweden; b University Clinic Primary Care, Skåne University Hospital, Region Skåne, Sweden; c Regional Cancer Centre South, Lund, Sweden; d Division of Oncology, Department of Clinical Sciences Lund, Lund University, Lund, Sweden; e Department of Hematology, Oncology and Radiation Physics, Skåne University Hospital, Lund, Sweden; f Division of Psychiatry, Department of Clinical Sciences Lund, Lund University, Lund, Sweden

**Keywords:** Cancer survivors, primary care, access to health care, patient satisfaction, surveys and questionnaires, Sweden

## Abstract

**Background:**

The number of cancer survivors is increasing, but their health care needs are often unmet. Increased involvement of primary care has been suggested to address this. Several barriers to satisfactory primary care contacts for cancer survivors have been identified but little studied in a Swedish context.

**Aim:**

To explore barriers to satisfactory primary care contacts for Swedish cancer survivors, and factors associated with these barriers.

**Method:**

In 2023, patients in southern Sweden diagnosed with colorectal, lung, breast, or prostate cancer 1–2 or 5–6 years ago were invited to complete a digital questionnaire. The questionnaire covered quality of life, health problems, health care needs, and health care contacts. Respondents also rated the extent to which they had experienced six types of barriers to satisfactory primary care contacts. Logistic regression models were fitted to explore the relationship between barriers and other questionnaire variables.

**Results:**

A total of 8262 individuals were invited, and 2131 responded. Two-thirds (1429/2131; 67%) reported one or more barriers to satisfactory primary care contacts. Lack of *staff continuity* was most prevalent, with 48% (1031/2131) experiencing a barrier, followed by lack of *access.* Survivors with additional comorbidities, more cancer-related health problems, problem-induced primary care contacts, and those who lacked a regular primary care physician had higher odds of perceiving barriers than their counterparts. This also applied to participants who did not have an active handover compared to those still in treatment.

**Conclusion:**

From the perspective of Swedish cancer survivors, improved relational continuity and access to primary care is needed.

## Background

An increasing proportion of the population live with or have survived cancer [1]. In 2020, there were 19 million new cases of cancer globally. In 2040, the global cancer incidence is estimated to reach 28 million cases [1]. With improved diagnostics, treatment and standards of living, more cancer patients live to become cancer survivors. In Sweden, the relative 5-year survival rate of all cancers combined has increased from around 40% in 1974–1978 to around 75% in 2019–2023 [2]. Most cancer survivors experience physical and psychological consequences of the cancer disease and its treatment in the short term and in the longer term [3–5]. Some of these consequences will result in an increased need for primary care [6]. However, previous international research has indicated that cancer survivors’ health care needs often are not fulfilled [3,7]. These unmet needs include physical as well as emotional and practical issues, in temporal proximity to the cancer diagnosis but also up to 10 years later [3,7].

Lack of cooperation between specialized cancer care and primary care has been pointed out as part of the problem in meeting cancer survivors’ needs, and increased involvement of primary care has been suggested [8,9]. Important barriers to integrated or shared cancer care have been identified in prior international studies [10–12]. These barriers center on communication between specialized cancer care and primary care, and on knowledge and training in primary care [10–12]. In Sweden, primary care is team-based and has an extensive mandate that includes managing acute and chronic conditions, general palliative care, rehabilitation, and prevention. At the same time, Swedish primary care receives a relatively low proportion of total health care spendings and has challenges regarding access and continuity [13–15]. Thus, it is reasonable to assume that internationally identified barriers to integrated cancer survivorship care will be at least as significant in a Swedish context. Nonetheless, to our knowledge, the role of primary care for Swedish cancer survivors has been insufficiently studied. In two prior qualitative studies, we therefore explored Swedish cancer survivors’ experience of primary care [16,17]. Several barriers to satisfactory primary care contacts were identified by the participants, including deficient coordination with specialized cancer care, lack of continuity, and low availability [16]. Similar barriers have to some extent been identified in other national contexts [8,12]. However, to our knowledge, no Swedish quantitative studies have been reported in this area.

More detailed understanding of barriers from the cancer survivors’ perspective may serve to improve primary care for this growing and potentially vulnerable patient group. Therefore, we set out to further explore barriers to satisfactory primary care contacts for Swedish cancer survivors, and cancer-related, health-related, and primary care-related factors associated with these barriers, through a survey study.

## Method

### Study design

This is a cross-sectional study using data from the Swedish Cancer Registry and data collected in a digital survey. The manuscript was prepared in adherence with the Strengthening the Reporting of Observational Studies in Epidemiology (STROBE) guidelines [18].

### Setting, participants, and data collection

Adult patients in the Swedish Southern Health Care Region (total population: 1.9 million) who were diagnosed with colorectal, lung, breast, or prostate cancer 1–2 years or 5–6 years prior to questionnaire distribution were identified *via* the Swedish Cancer Registry in 2023. All identified patients (*n* = 8262) were invited *via* postal mail to fill out a digital questionnaire using the Research Electronic Data Capture (REDCap) platform [19,20]. The questionnaire covered background factors, quality of life, health problems, health care needs, and health care contacts (Supplement 1). We included existing instruments in the form of the 19-item version of the Assessment of Rehabilitation Needs Checklist (ARNC), the European Organization for Research and Treatment of Cancer Quality of Life Questionnaire (EORTC QLQ-C30) version 3, and one item on self-rated health from the 36-Item Short Form Health Survey (SF-36) as well as items based on our prior qualitative studies [16,17,21–25]. Patient representatives were consulted regarding content, relevance, and wording during the development, but no formal pilot testing was done.

### Variables and statistical methods

**Figure 1.**
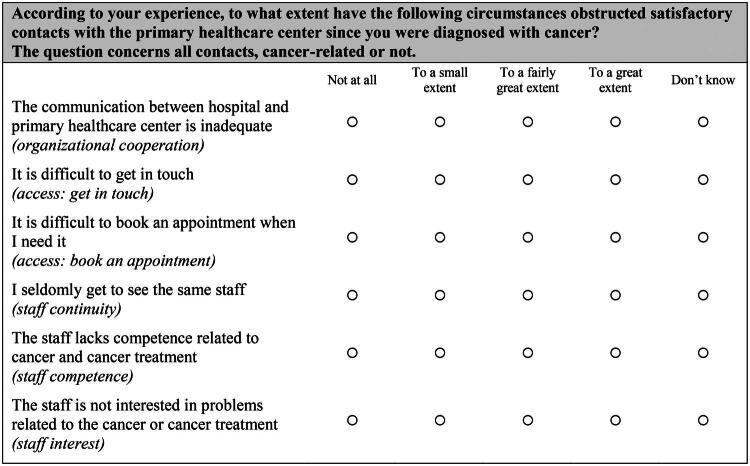
Questionnaire item regarding barriers to satisfactory primary care contacts for cancer survivors. Italicized text in parentheses is used to refer to the item in-text and was not part of the questionnaire.

This study describes and analyzes the questionnaire responses focusing on barriers to satisfactory primary care contacts. The questionnaire item regarding barriers (Supplement 1, item 31) was constructed based on our prior qualitative studies, and asks respondents to rate the extent to which they have experienced that six different circumstances have obstructed satisfactory primary care contacts since they were diagnosed with cancer (Figure 1) [16,17]. For analysis, ratings were dichotomized into the absence of a barrier (rating ‘not at all’ or ‘don’t know’) or the presence of a barrier (to any extent, i.e. rating ‘to a small extent’, ‘to a fairly great extent’, or ‘to a great extent’). This was done for each sub-item (each barrier), and also for all sub-items combined to obtain a measure of whether participants had perceived any barrier.

The following variables were analyzed regarding association with the perception of barriers to satisfactory primary care contacts, and the results were used to inform the selection of variables for further analysis (item numbers refer to the questionnaire, Supplement 1):

Background variables: Age (years), sex (man/woman), native language (Swedish/other). Education (item 5) was dichotomized into high school or lower (none, compulsory school, or senior high school) and post-high school.
Cancer-related variables: Time since diagnosis (1–2/5–6 years), type of cancer (prostate/breast/colorectal/lung).
Health-related variables: Self-rated health (item 10) was used as an overall measure of health (poor/fair/good/very good/excellent) and was also dichotomized into low (poor/fair) or high (good/very good/excellent). The EORTC QLQ-C30 physical function scale (score 0–100, calculated from 5 items) was used as a measure of overall physical function. Total number of cancer-related health problems reported in the ARNC (0–19) was included as a measure of needs, and was also divided according to distribution into approximate thirds of the sample (0–2/3–5/6+ problems).
Primary care-related variables: Number of additional comorbidities common to primary care (item 23) had a total of 11 options and answers were categorized as 0/1/2/3+ comorbidities. Primary care center operator (item 26) was dichotomized as public or private, and response ‘don’t know’ was excluded. Knowledge of a regular primary care physician (item 27) was dichotomized as yes or no (‘no’ or ‘don’t know’). For satisfaction with primary care contacts since cancer diagnosis (item 30), all ratings were used (very poorly/poorly/neutral/well/very well). Post-diagnosis cancer-related primary care contacts (item 22) were divided into three types depending on patient-reported reasons: routine only (for blood sampling or assistance with aids), problem-induced (for physical, psychological/social or other problems), or no cancer-related primary care contact. An active handover (item 28, opened if response: ‘no’ on item 14 regarding ongoing/planned cancer treatment) was defined as reporting of referral from specialized cancer care to primary care after conclusion of treatment and was dichotomized as yes or no (‘no’ or ‘don’t know’). For regression analysis, we constructed a variable to indicate care pathway position, categorizing participants in ongoing/planned cancer treatment (‘yes’ on item 14) as well as participants who had concluded their treatment (‘no’ on item 14). The latter group was further categorized depending on reporting of active handover (‘yes’, ‘no’, or ‘don’t know’ on item 28).

We performed a complete case analysis, however in the submitted questionnaires there was no missing data to address. Pearson’s chi-square test was used to analyze the association between categorical variables and ratings on barriers. As prerequisites for parametric analysis were not fulfilled, Kruskal Wallis test was used for continuous variables. Post-hoc tests were performed using the Bonferroni correction. Spearman’s rank-order correlation coefficient was calculated to measure the strength of association between ratings on barriers and on satisfaction with primary care contacts. Logistic regression models were fitted to further explore the relationship between the included variables and perceived barriers to satisfactory primary care contacts. The selection of variables was based on theoretical relevance and on the results of bivariate analyses. As a sensitivity analysis, we excluded the participants that rated ‘don’t know’ on each barrier in the logistic regression models.

### Ethics

The project has been approved by the Swedish Ethical Review Authority (dnr 2022-05569-01) and a regional consent for the retrieval of patient register data (KVB 358-22). Participation was voluntary, and all participants consented to participate after receiving study information. Data was pseudonymized and handled in accordance with data security regulations.

## Results

A total of 8262 individuals were invited, and 2131 responded resulting in a response rate of 26%. Background characteristics and response rates are presented in Table 1.

**Table 1.** Background characteristics and response rates for our 2023 survey of Southern Swedish cancer survivors’ rehabilitation needs and health care contacts (*N* = 2131).

Variable	Participants, *n* (%)	Response rate^a^, %
Sex		
Women	935 (44)	25
Men	1196 (56)	27
Age (years)		
18–49	146 (7)	34
50–59	327 (15)	36
60–69	644 (30)	33
70–79	817 (38)	26
≥80	197 (9)	12
Time since diagnosis		
1–2 years	1278 (60)	27
5–6 years	853 (40)	24
Type of cancer		
Prostate	906 (43)	27
Breast	720 (34)	27
Colorectal	405 (19)	24
Lung	100 (5)	17
Native language Swedish^b^	1997 (94)	
Post-high school education^b^	1101 (52)	
Total	2131 (100)	26

^a^ Out of 8262 individuals invited.

^b^ Response rate cannot be calculated as data were obtained from the questionnaire.

Participant ratings on barriers to satisfactory primary care contacts after cancer diagnosis are provided in Tables 2 and 3. *Staff continuity* had the highest proportion of participants perceiving this as a strong barrier (341/2131; 16%) and as a barrier to any extent (1031/2131; 48%).

**Table 2.** Distribution of barriers to satisfactory primary care contacts perceived by Southern Swedish cancer survivors (*N* = 2131).

Perceived barrier	To what extent? (*n*, %)				
	Don’t know	Not at all	To a small extent	To a fairly great extent	To a great extent
Organizational cooperation	823 (38.6)	691 (32.4)	256 (12.0)	158 (7.4)	203 (9.5)
Access: get in touch	223 (10.5)	1068 (50.1)	482 (22.6)	205 (9.6)	153 (7.2)
Access: book an appointment	241 (11.3)	1041 (48.9)	442 (20.7)	224 (10.5)	183 (8.6)
Staff continuity	247 (11.6)	853 (40.0)	418 (19.6)	272 (12.8)	341 (16.0)
Staff competence	981 (46.0)	765 (35.9)	182 (8.5)	107 (5.0)	96 (4.5)
Staff interest	819 (38.4)	950 (44.6)	158 (7.4)	96 (4.5)	108 (5.1)

**Table 3.** Distribution of barriers (dichotomized) to satisfactory primary care contacts perceived by Southern Swedish cancer survivors (*N* = 2131).

Perceived barrier	To what extent? (*n*, %)	
	Absence of barrier^a^	Presence of barrier to any extent^b^
Organizational cooperation	1514 (71.0)	617 (29.0)
Access: get in touch	1291 (60.6)	840 (39.4)
Access: book an appointment	1282 (60.2)	849 (39.8)
Staff continuity	1100 (51.6)	1031 (48.4)
Staff competence	1746 (81.9)	385 (18.1)
Staff interest	1769 (83.0)	362 (17.0)

^a^ Rating ‘don’t know’ or ‘not at all’.

^b^ Rating ‘to a small extent’, ‘to a fairly great extent’, or ‘to a great extent’.

*Access*, in terms of difficulty to *get in touch* and to *book an appointment*, appeared as the second largest barrier considering ratings to any extent. The two items on access had similar ratings with 39% (840/2131) and 40% (849/2131), respectively, perceiving a barrier to any extent. For the three remaining barriers (*organizational cooperation*, *staff competence*, and *staff interest*), a high proportion of participants rated ‘don’t know’ (38–46%) (Tables 2 and 3).

**Figure 2.**
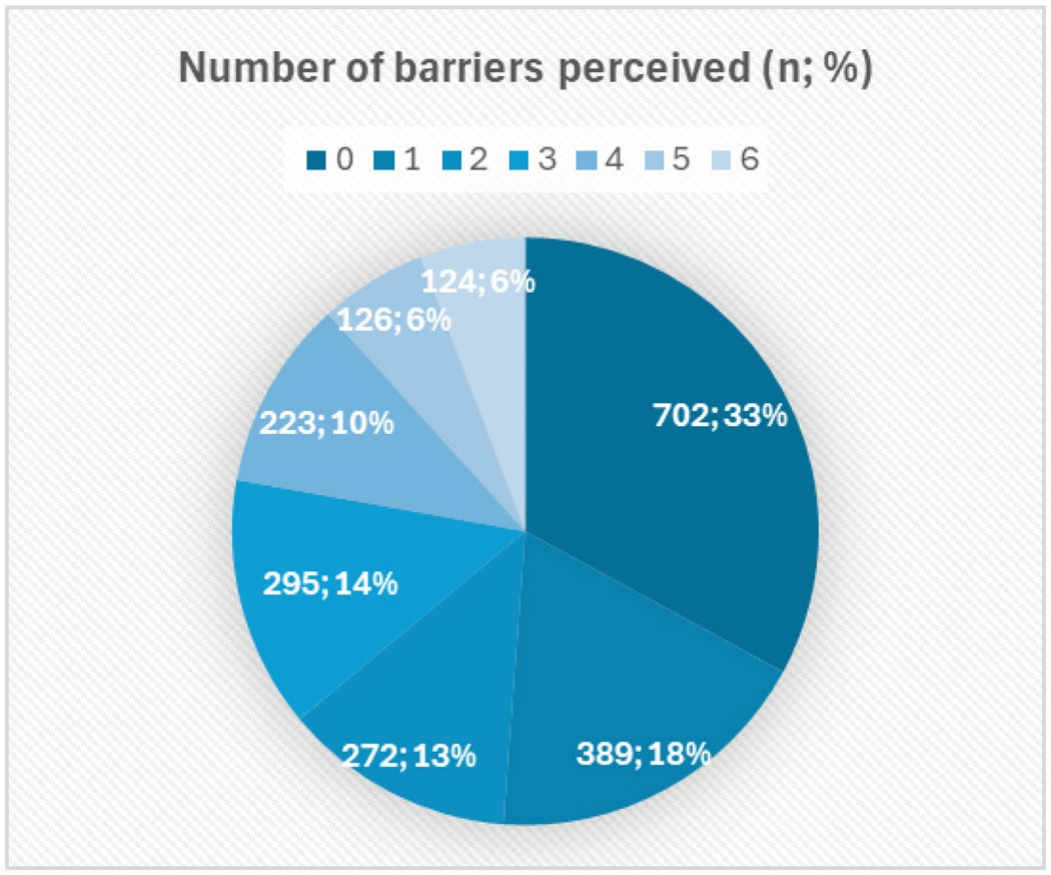
Number of barriers to primary care contacts perceived by southern Swedish cancer survivors (*N* = 2131). Participants rated the degree to which each of six circumstances (*organizational cooperation*, *access: get in touch*, *access: book an appointment*, *staff continuity*, *staff competence*, and *staff interest*) had obstructed satisfactory primary care contacts. The figure visualizes how many barriers were perceived, regardless of extent.

Figure 2 illustrates whether barriers were perceived regardless of type and extent. Most participants (1429/2131; 67%) experienced one or more barriers. It was more common to experience barriers in one to three areas than in four to six areas (Figure 2).

The different barriers showed similar strengths of association with ratings on satisfaction with primary care contacts after cancer diagnosis (Table 4). *Staff continuity* showed the weakest association (*ρ* = 0.43, *p* < .001) while *organizational cooperation* showed the strongest association (*ρ* = 0.58, *p* < .001).

**Table 4.** Strength of association between perceived barriers and satisfaction with primary care contacts for Southern Swedish cancer survivors (*N* = 2131).

Perceived barrier^a^	Satisfaction^b^	
	Spearman’s *ρ*	*p*-value
Organizational cooperation (*n* = 1123)	0.58	<.001
Access: get in touch (*n* = 1515)	0.53	<.001
Access: book an appointment (*n* = 1506)	0.52	<.001
Staff continuity (*n* = 1514)	0.43	<.001
Staff competence (*n* = 1017)	0.54	<.001
Staff interest (*n* = 1162)	0.55	<.001

^a^ Ratings coded for analysis: Not at all = 1, to a small extent = 2, to a fairly great extent = 3, to a great extent = 4. Response ‘don’t know’ was excluded from analysis.

^b^ Ratings coded for analysis: Very well = 1, well = 2, neutral = 3, poorly = 4, very poorly = 5. Response ‘don’t know’ was excluded from analysis.

The distribution of participant characteristics, and cancer-, health-, and primary care-related variables in relation to the perception of each barrier is detailed in Supplementary Tables 1-6. For a broader measure, we also included the presence of any barrier (Supplementary Table 7). The results of logistic regression analysis, using the presence or absence of the different barriers as the outcome, are shown in Table 5. Cancer survivors with a higher number of cancer-related health problems according to the ARNC (6+) perceived more barriers of all types than those with a lower number of problems (0–2). The association was most evident for *organizational cooperation* (OR 2.6, 95% CI 2.0–3.4). Participants with additional comorbidities (2+ vs 0) also experienced more barriers of all types. Further, those with problem-induced cancer-related primary care contacts reported more barriers than those with routine contacts only. The overall perception of barriers was also higher for cancer survivors who did not have an active handover from specialized cancer care to primary care compared to those still in cancer treatment. In addition, lacking a regular primary care physician was strongly associated with experiencing *staff continuity* as a barrier (OR 4.2, 95% CI 3.4–5.1), but also with perceptions of *access* (OR 2.0, 95% CI 1.7–2.4; OR 1.6, 95% CI 1.3–1.9). Finally, cancer survivors with low self-rated health, non-native Swedish speakers, and women appeared to perceive more barriers than their counterparts, while those with high school education or lower perceived less barriers than those with higher education, though associations were not as strong as for the other factors (Table 5).

**Table 5.** Logistic regression models^a^ of factors associated with the perception of barriers to satisfactory primary care contacts for Southern Swedish cancer survivors (*N* = 2131).

	Perceived barrier to satisfactory primary care contacts, OR (95% CI)						
Variables	Organizational cooperation^b^	Access:get in touch^c^	Access:book an appointment^d^	Staff continuity^e^	Staff competence^f^	Staff interest^g^	Any barrier^h^
Age (years)	1.0 (0.98–1.000)	1.0 (0.999–1.02)	1.0 (0.999–1.02)	1.0 (0.996–1.02)	1.0 (0.97–0.996)	1.0 (0.99–1.02)	1.0 (0.99–1.01)
Sex							
Man	Ref.	Ref.	Ref.	Ref.	Ref.	Ref.	Ref.
Woman	1.0 (0.8–1.3)	1.4 (1.1–1.7)	1.4 (1.2–1.7)	1.2 (1.01–1.5)	1.5 (1.1–1.9)	1.5 (1.2–2.0)	1.3 (1.03–1.6)
Native language							
Swedish	Ref.	Ref.	Ref.	Ref.	Ref.	Ref.	Ref.
Other	1.4 (0.9–2.0)	1.4 (.98–2.1)	1.7 (1.2–2.4)	1.6 (1.1–2.3)	0.9 (0.6–1.5)	1.3 (0.8–2.0)	1.6 (1.04–2.5)
Education							
Post-high school	Ref.	Ref.	Ref.	Ref.	Ref.	Ref.	Ref.
High school or lower	0.7 (0.6–0.9)	0.8 (0.6–0.9)	0.8 (0.7–0.9)	0.95 (0.8–1.1)	0.9 (0.7–1.1)	0.8 (0.6–1.05)	0.8 (0.7–0.99)
Number of comorbidities							
0	Ref.	Ref.	Ref.	Ref.	Ref.	Ref.	Ref.
1	0.9 (0.7–1.2)	1.1 (0.9–1.4)	1.1 (0.9–1.4)	1.3 (1.1–1.7)	0.9 (0.7–1.2)	0.9 (0.7–1.3)	1.2 (0.95–1.5)
2	1.6 (1.2–2.1)	1.6 (1.2–2.2)	1.7 (1.3–2.3)	2.1 (1.6–2.7)	1.2 (0.8–1.7)	1.7 (1.2–2.5)	2.2 (1.6–2.9)
3+	1.8 (1.3–2.5)	1.9 (1.4–2.6)	1.9 (1.4–2.6)	1.9 (1.4–2.6)	1.5 (1.1–2.3)	1.9 (1.3–2.8)	1.8 (1.3–2.5)
Self-rated health							
Good to excellent	Ref.	Ref.	Ref.	Ref.	Ref.	Ref.	Ref.
Fair to poor	1.4 (1.1–1.8)	1.2 (0.96–1.6)	1.5 (1.2–1.9)	1.0 (.0.8–1.3)	1.1 (0.8–1.5)	1.2 (0.9–1.6)	1.5 (1.1–2.0)
ARNC total number of problems							
0–2	Ref.	Ref.	Ref.	Ref.	Ref.	Ref.	Ref.
3–5	1.8 (1.4–2.4)	1.3 (1.1–1.7)	1.3 (0.99–1.6)	1.2 (0.9–1.5)	1.8 (1.3–2.5)	1.2 (0.9–1.7)	1.5 (1.2–1.9)
6+	2.6 (2.0–3.4)	1.9 (1.5–2.4)	2.0 (1.6–2.5)	1.8 (1.4–2.3)	2.4 (1.7–3.3)	2.3 (1.7–3.1)	2.5 (2.0–3.3)
Cancer-related primary care contacts							
Routine contacts only	Ref.	Ref.	Ref.	Ref.	Ref.	Ref.	Ref.
Problem-induced contacts	2.0 (1.6–2.6)	1.5 (1.2–2.0)	1.4 (1.1–1.8)	1.4 (1.1–1.8)	2.0 (1.5–2.6)	1.8 (1.3–2.4)	1.7 (1.3–2.3)
No cancer-related contacts	0.9 (0.7–1.2)	0.9 (0.7–1.1)	1.1 (0.8–1.4)	1.2 (0.9–1.5)	0.8 (0.6–1.1)	0.8 (0.6–1.1)	1.0 (0.8–1.3)
Regular primary care physician							
Yes	Ref.	Ref.	Ref.	Ref.	Ref.	Ref.	Ref.
No	1.0 (0.8–1.2)	2.0 (1.7–2.4)	1.6 (1.3–1.9)	4.2 (3.4–5.1)	1.3 (0.98–1.6)	1.3 (1.0–1.7)	2.0 (1.6–2.4)
Care pathway position							
Ongoing/planned cancer treatment	Ref.	Ref.	Ref.	Ref.	Ref.	Ref.	Ref.
Active handover: Yes	1.1 (0.8–1.6)	1.3 (0.9–1.8)	1.3 (0.9–1.9)	1.4 (0.997–2.0)	1.0 (0.6–1.5)	1.0 (0.7–1.6)	1.3 (0.9–1.9)
Active handover: No	1.7 (1.3–2.3)	1.6 (1.2–2.1)	1.7 (1.3–2.2)	1.4 (1.1–1.9)	1.2 (0.9–1.6)	1.5 (1.1–2.1)	1.4 (1.03–1.8)
Active handover: Don’t know	0.8 (0.6–1.1)	1.4 (1.04–1.8)	1.3 (.99–1.7)	1.4 (1.1–1.9)	0.7 (0.5–1.03)	1.1 (0.7–1.6)	1.3 (0.95–1.7)

^a^Variables in models: age, sex, native language, number of comorbidities (0–3+), self-rated health, Assessment of Rehabilitation Needs Checklist (ARNC) total number of problems, cancer-related primary care contacts, regular primary care physician, care pathway position.

^b^Nagelkerke R^2^ = 0.16; Hosmer-Lemeshow test X^2^ = 8.2, *p* = 0.42.

^c^Nagelkerke R^2^ = 0.12; Hosmer-Lemeshow test X^2^ = 8.6, *p* = 0.38.

^d^Nagelkerke R^2^ = 0.12; Hosmer-Lemeshow test X^2^ = 7.3, *p* = 0.51.

^e^Nagelkerke R^2^ = 0.18; Hosmer-Lemeshow test X^2^ = 11.4, *p* = 0.18.

^f^Nagelkerke R^2^ = 0.13; Hosmer-Lemeshow test X^2^ = 3.3, *p* = 0.91.

^g^Nagelkerke R^2^ = 0.12; Hosmer-Lemeshow test X^2^ = 6.5, *p* = 0.59.

^h^Nagelkerke R^2^ = 0.15; Hosmer-Lemeshow test X^2^ = 6.4, *p* = 0.60.

Sensitivity analyses excluding response ‘don’t know’ on each barrier in the logistic regression models did not change the overall results (data not shown).

## Discussion

### Main findings

This cross-sectional survey explored Swedish cancer survivors’ perceptions of barriers with regards to primary care contacts 1–2 years and 5–6 years after cancer diagnosis. Two-thirds of the participants reported one or more barriers. Lack of *staff continuity* and lack of *access* were most prevalent. Participants with more comorbidities, more cancer-related health problems, problem-induced primary care contacts, and those who lacked a regular primary care physician had higher odds of perceiving barriers than their counterparts. This also applied to survivors who did not have an active handover from specialized cancer care to primary care compared to those still in cancer treatment.

### Strengths and limitations

A strength of this study is the use of recently collected extensive questionnaire data covering multiple aspects of participant characteristics and experience. The questionnaire was developed based on prior studies with cancer survivors, and patients were included in the development process [16,17]. Moreover, all cancer survivors in a large part of southern Sweden were invited to provide their views. Thus, measures have been taken to explore patient views in-depth. The questionnaire also contained several validated parts such as the EORTC QLQ-C30 and the ARNC. However, the complete version of the questionnaire was not pilot tested, which means that reliability and validity are partially uncertain. There appeared to be a floor effect regarding the perception of barriers on all items (32–50% rated ‘not at all’), but a ceiling effect only on ‘staff continuity’ (16% rated ‘to a great extent’). This indicates that the questionnaire could sufficiently distinguish between the different degrees of perceived barriers to satisfactory primary care contacts.

A considerable proportion of participants refrained from providing an opinion (i.e. rated ‘don’t know’) on barriers other than *staff continuity* and *access*. This could be interpreted as participants not experiencing a barrier in the other requested areas but is still a limitation. For analysis, we dichotomized ratings into the presence or absence of a barrier. Here, we classified the response ‘don’t know’ as indicating that the patient did not experience a significant barrier and grouped it together with the rating’ not at all’ to form ‘absence of barrier’. This choice increased the amount of data that could be analyzed but also the risk for misclassification. Nonetheless, sensitivity analyses excluding the ‘don’t know’ responses did not change the overall results.

We included cancer survivors diagnosed 1–2 years or 5–6 years prior to questionnaire distribution to capture experiences early as well as later in the cancer continuum and to discern any differences between these patient groups. However, this decreases generalizability to cancer survivors at other points in time after diagnosis. Another possible weakness of this study was the participation rate (2131/8262; 26%). Those older than 70 years and lung cancer survivors had a lower response rate. Thus, there may be a non-response bias which decreases generalizability to older cancer survivors and to lung cancer survivors. In addition, as invitation was limited to those diagnosed with lung, colorectal, breast, and prostate cancer, our results may not be generalizable to survivors from other types of cancers. Further, non-native Swedish speakers were under-represented, with 6% in the study sample compared to slightly more than 20% foreign-born in the Swedish population [26].

Considering care pathway position, using logistic regression analysis, we compared the perception of barriers to satisfactory primary care contacts between participants still in cancer treatment (reference group) and those who had finished their treatment with or without an active handover to primary care. This allowed for conclusions regarding active handover status compared to still being in treatment, but not for direct comparison between active handover and no active handover. It should also be noted that this was a cross-sectional survey; thus, associations can be studied but no conclusions can be made in terms of causation. Finally, we investigated barriers to satisfactory primary care contacts as identified in our prior qualitative studies and in the scientific literature, and we thus cannot make conclusions regarding other possible barriers. Nonetheless, our overall measure of barriers—the presence of any barrier—may serve as an indication.

### Findings in relation to prior research

The cancer survivors in our study rated lack of *staff continuity* and *access* as the most frequent barriers to satisfactory primary care contacts. These are well-known problems in Swedish primary care, and do not only pertain to cancer survivors [13–15]. In the 2023 International Health Policy Survey, only 32% of Swedish participants reported that they had a regular primary care physician in comparison with the total average of 81% [14]. The benefits of primary care continuity, including on reducing emergency unit visits and mortality rates, have been demonstrated in Sweden and internationally [27,28]. Our results further emphasize the importance of continuity and of timely access for patients with a need for primary care, adding the perspective of cancer survivors. Interestingly, ratings on *staff interest* and *staff competence* suggest that these areas were rarely perceived as a barrier to satisfactory primary care contacts by the cancer survivors in our study. These findings to some extent contradict findings in our previous qualitative studies and in other contexts, where primary care staff competence is often put forth as a problem area when it comes to cancer survivorship care [8,10,12,16]. However, the high proportion of participants in our study that refrained from providing an opinion on these areas (38% and 46%) limits any conclusions. As access was reported as a frequent barrier (40%), participants may not have been given the opportunity to see a health care professional at the primary health care center and thus nor to evaluate their interest or competence.

Even if the range was relatively narrow, it may be noted that *staff continuity* showed the weakest correlation with ratings on satisfaction with primary care contacts (*ρ* = 0.43), while perceptions of *organizational cooperation* showed the strongest correlation (*ρ* = 0.58). Prior studies have generally focused more on organizational factors than on patient experience, and these studies have also reported measures comparable to *organizational cooperation* as a barrier to the successful provision of survivorship care [8,12]. A frequent suggestion to address this barrier is the creation of survivorship care plans in specialized cancer care which are then transferred to primary care, as a means of planning and communication between the different levels of care but also to include the patient in the process [8,10–12]. Survivorship care plans have, however, been difficult to implement [8].

According to Ronald M. Andersen’s Behavioral Model of Health Services Use, factors affecting the perception of barriers to functioning health care contacts could be divided into mutable and non-mutable [29]. Considering the factors primarily identified in our logistic regression analyses, number of comorbidities (predisposing characteristics) and cancer-related health problems (needs) can be considered to have low mutability in themselves [29]. However, these barriers could still be countered by prioritizing patients with more comorbidities and more problems. This, in turn, could be done through addressing the major mutable factors identified in our analysis. That is, by enabling problem-induced primary care contacts through active handover (referral) from specialized cancer care to primary care, where a referral should include comorbidities and health care needs. This would primarily address barriers due to lack of *organizational cooperation* but could thus facilitate *access* and *staff continuity*—even if policy action is also needed [8,10,13,15,29].

## Conclusions

In conclusion, our results emphasize the importance of improved relational continuity and access to primary care from the perspective of cancer survivors. Active handovers from specialized cancer care to primary care could be one way to facilitate this. Such handovers may focus on patients with additional comorbidities, more cancer-related health problems, and on decreasing and facilitating problem-induced health care contacts. Future research may include an intervention focusing on these areas for further evaluation.

## Supplementary Material

Supplement 1 Questionnaire.pdf

STROBE checklist cross sectional.docx

## Data Availability

The data that support the findings of this study are restricted by the Swedish Ethical Review Authority in order to protect participants’ privacy.
