# Supplementary material for: Barriers to satisfactory primary care contacts for Swedish cancer survivors: a cross-sectional survey
Source: Scand J Prim Health Care. 2026 Jul 11;44(1):2701788. doi: 10.1080/02813432.2026.2701788 (PMC13360504; doi:10.1080/02813432.2026.2701788)
Supplement: Supplement 1 Questionnaire.pdf [file IPRI_A_2701788_SM6052.pdf]

The role of primary care in caring for people with cancer in the Southern healthcare region: A survey study

[NB! This is a paper representation of the digital questionnaire, translated from the original Swedish.]

Below you find a number of questions that we want you to answer about yourself, your health and your healthcare contacts. To begin with, we ask for your consent. It is important that you have read the information that was enclosed with the invitation (also available in digital form through the link immediately below). You are also welcome to contact us in any of the ways described in the information, in case you have further questions. Please note that the questionnaire is dynamic, which means that new questions might open depending on your previous answers.

[link to study information]

1. I Have received written information about the study, have been given time to consider it and the opportunity to ask questions to the project group. I am allowed to keep the written information I have been sent.
   I consent to participation in the study The role of primary care in caring for people with cancer in the Southern healthcare region: A survey study, and to my personal information being handled in the way described in the study information.

   ☐ Yes

A few questions about you

2. Age (in full years) ________________

3. Sex

   ☐ Man

   ☐ Woman

   ☐ Other/decline to disclose

4. Is Swedish your native language?

   ☐ Yes

   ☐ No

5. Which is the highest education that you have ***completed***?

   ☐ None

☐ Compulsory school

☐ Senior high school (two or three years)

☐ Post-senior high school (university)

6. Describe your household.

☐ Single

☐ Cohabitation (married, partner, cohabitant).

☐ Other (e.g., three-generation household, cohabitation with friends, collective household)

7. Are you the legal guardian of one or more children?

☐ Yes

☐ No

8. What is the estimated travel time from your home to the primary healthcare center where you are listed, by the means of transportation that you would normally use?
*Please provide answer in **whole minutes*** ____________________

9. If you were to become acutely ill, what is the estimated travel time from your home to the closest hospital, by the means of transportation that you would normally use?
*Please provide answer in **whole minutes*** ____________________

Your health

10. In general, would you say your health is:

☐ Excellent

☐ Very good

☐ Good

☐ Fair

☐ Poor

11. Are you on sick leave (full or part time)?

☐ Yes

☐ No

12. [*If yes on Q11*] Is the sick leave related to cancer?

☐ Yes

☐ No

13. What kind(s) of cancer treatment have you undergone?
Please check all choices that apply to you.

☐ Surgery

☐ Radiotherapy

☐ Chemotherapy

☐ Hormonal treatment

☐ Other pharmaceutical treatment

☐ None

☐ Do not know

14. Are you currently undergoing, or are you planned for, cancer treatment?

☐ Yes

☐ No

15. [*If yes on Q14*] What is the purpose of the current or planned treatment?

☐ Curative or preventive

☐ Control of cancer growth or symptom relief

☐ Do not know

16. [*If no on Q14*] What has been the purpose(s) of treatments that you have undergone before?
Please check all choices that apply to you.

☐ Curative or preventive

☐ Control of cancer growth or symptom relief

☐ Do not know

Your quality of life

Below are 30 questions that concerns your quality of life.

"Medical treatment" is to be understood as the medical treatment of your cancer.

17. [*Please refer to EORTC-QLQ-C30 ver. 3*].

Your cancer-related needs and healthcare contacts

Below you find questions about which needs you have experienced related to your (current or previous) cancer ***during the last month***. If you indicate that you have been affected by a certain problem, we will ask follow-up questions about healthcare needs and contacts.

18. [*Please refer to ACRN ver. 2 (Nevo-Ohlsson & Alkebro 2016)*].

19. [*If any problem was indicated in response to Q18*] ***During the last month***, have you experienced a need to contact healthcare services for any of the problems you have experienced?
Please check all choices that apply to you. If you have not experienced any need for contact for a particular problem, leave both boxes empty.
[*Answered separately for each individual problem indicated in response to Q18*]

☐ Yes, the hospital

☐ Yes, the primary healthcare center

20. [*If any need for primary care contacts was indicated in response to Q19*] To what extent do you experience that contacts with the primary healthcare center (physical, phone or digital) have helped you with the problem or problems?
[*Answered separately for each individual problem associated with a need for contacts with the primary healthcare center in response to Q19*]

☐ Has not been in contact

☐ To a small extent

☐ To a fairly great extent

☐ To a great extent

☐ Do not know

21. [*If Q19 = need for hospital contacts indicated*] To what extent do you experience that contacts with the hospital (physical, phone or digital) have helped you with the problem or problems?
[*Answered separately for each individual problem indicated to be associated with a need for contacts with the hospital in response to Q19*]

☐ Has not been in contact

☐ To a small extent

☐ To a fairly great extent

☐ To a great extent

☐ Do not know

Your primary care contacts

22. Since you were diagnosed with cancer, have you been in contact with the primary healthcare center for reasons associated with the cancer disease or its treatment?
Please check all choices that apply to you.

☐ Yes, for blood sampling

☐ Yes, for assistance with aids (e.g., PICC-line, ostomy, catheter)

☐ Yes, due to physical problems associated with cancer

☐ Yes, due to psychological or social problems associated with cancer

☐ Yes, for other reasons associated with cancer

☐ No

23. Do you suffer from any of the following chronic conditions or problems?
Please check all choices that apply to you.
The listed problems are not necessarily associated with cancer. The reason we ask is that they represent common reasons for contacts with primary care.

☐ High blood pressure

☐ Cardiovascular disease (e.g. myocardial infarction, heart failure, atrial fibrillation)

☐ Diabetes

☐ COPD (Chronic obstructive pulmonary disease)

☐ Asthma

☐ Thyroid disease

☐ Dyspepsia that requires daily medication

☐ IBS (Irritable bowel syndrome)

☐ Pain that you have regularly medicated during at least three months

☐ Anxiety or depression

☐ Other chronic illness (other than cancer)

24. [*If any condition was indicated in response to Q23*] Are you in regular contact with your primary healthcare center because of any problems indicated in the previous question?

☐ Yes

☐ No

25. [*If yes on Q24*] Who have you been seeing?
Please check all choices that apply to you.

☐ Physician

☐ Nurse/assistant nurse

☐ Social worker/psychologist/psychotherapist

☐ Dietician

☐ Physiotherapist/occupational therapist

☐ Other profession/do not know

26. Is the primary healthcare where you are listed publicly or privately operated?

☐ Publicly

☐ Privately

☐ Do not know

27. Do you have a regular physician at the primary healthcare center?

☐ Yes

☐ No

☐ Do not know

28. [*If no on Q14*] When primary cancer treatment concluded, were you actively handed over to the primary healthcare center, through referral?

☐ Yes

☐ No

☐ Do not know

29. How do you feel that the communication between the primary healthcare center and cancer specialist care has worked *since you were diagnosed with cancer*?

☐ Very well

☐ Well

☐ Neither well nor poorly

☐ Poorly

☐ Very poorly

☐ Do not know/no contacts

30. How well do you feel that your contacts with the primary healthcare center has worked *since you were diagnosed with cancer*?

☐ Very well

☐ Well

☐ Neither well nor poorly

☐ Poorly

☐ Very poorly

☐ Do not know/no contacts

31. *According to your experience*, to what extent have the following circumstances obstructed satisfactory contacts with the primary healthcare center since you were diagnosed with cancer?
The question concerns all contacts, cancer-related or not.

The communication between hospital and primary healthcare center is inadequate.

☐ Not at all

☐ To a small extent

☐ To a fairly great extent

☐ To a great extent

☐ Do not know

It is difficult to get in touch.

☐ Not at all

☐ To a small extent

☐ To a fairly great extent

☐ To a great extent

☐ Do not know

It is difficult to book an appointment when I need it.

☐ Not at all

☐ To a small extent

☐ To a fairly great extent

☐ To a great extent

☐ Do not know

I seldomly get to see the same staff.

☐ Not at all

☐ To a small extent

☐ To a fairly great extent

☐ To a great extent

☐ Do not know

The staff lacks competence related to cancer and cancer treatment

☐ Not at all

☐ To a small extent

☐ To a fairly great extent

☐ To a great extent

☐ Do not know

The staff is not interested in problems related to the cancer or cancer treatment

☐ Not at all

☐ To a small extent

☐ To a fairly great extent

☐ To a great extent

☐ Do not know

32. Do you have a close one that would be in need of support from healthcare services (e.g., counseling) because of the cancer that you have been diagnosed with?

☐ Yes

☐ No

☐ Do not know

33. [*If yes on Q32*] To your awareness, have they been offered support?

☐ Yes

☐ No

☐ Do not know

---

34. If we were to follow-up the individuals who have participated in this survey, would it be acceptable if we contacted you again?

☐ Yes

☐ No

When you are satisfied with your responses, click the **Submit** button to submit them. You have reached the end of this questionnaire.
